# Severe Spontaneous Pneumomediastinum in a Girl with Cystic Fibrosis

**DOI:** 10.1055/s-0041-1731274

**Published:** 2021-10-01

**Authors:** Csaba Zsiborás, Mária Adonyi, József Stankovics, András Farkas, Peter Vajda, Barnabás Rózsai

**Affiliations:** 1 Department of Pediatrics, University of Pécs, Medical School, Pecs, Hungary

**Keywords:** spontaneous pneumomediastinum, cystic fibrosis, mediastinal drainage

## Abstract

We report on an 11-year-old girl with cystic fibrosis who presented with thoracic pain and an extensive subcutaneous emphysema and subsequently developed progressive respiratory distress. The chest computed tomography revealed a huge pneumomediastinum. Due to the development of severe respiratory failure, urgent needle thoracocentesis was necessary that resulted in only temporary improvement. Therefore, under general anesthesia two mediastinal drains were introduced. Using active suction, the size of the pneumomediastinum decreased gradually and the drains were removed after 3 weeks. Here, we describe an extremely rare situation, when acute surgical intervention was necessary in a child with spontaneous pneumomediastinum.

## Introduction

Pneumomediastinum is a rare condition in childhood. It can develop spontaneously; sometimes it is associated with underlying disorders or conditions (such as asthma, cough, intraoperative injuries of the trachea or esophagus, foreign body aspiration, and penetrating chest trauma). 1 2 Usually, it does not lead to significant complications and resolves spontaneously. In special circumstances (e.g., blunt trauma), the mediastinal pressure can increase significantly and rarely it leads to compression of the great vessels and the airways, leading to a life-threatening situation. 3 In such cases, an urgent thoracotomy is necessary.

The symptoms of spontaneous pneumomediastinum (SPM) may include chest pain and dyspnea and signs include subcutaneous emphysema and quiet heart sounds. Chest X-ray can help to confirm the suspected diagnosis.

Fitzwater et al reported almost 100 children with SPM, none of them required thoracic drainage or any other surgical interventions. In this cohort, in the development of SPM, asthma was the main precipitating factor (∼40%). 1

Cystic fibrosis (CF) involves multiple organ systems, especially the lung with recurrent and chronic airway infections. This can lead to bronchiectasis or pneumothorax (PTX). PTX appears in 3.4% of patients with CF during their lifetime. 4 Only a few papers are available in the literature, where pneumomediastinum are mentioned as a complication of CF. 4

Moreover, the association of the two entities above (SPM and CF) are extremely rare, especially, when surgical intervention was required.

We present the case of a girl diagnosed with CF who suddenly developed a life-threatening SPM and required emergency thoracic/mediastinal intervention and a sustained surgical drainage. Her family agreed to publish the case and pictures.

## Case Report

**Fig. 1:**
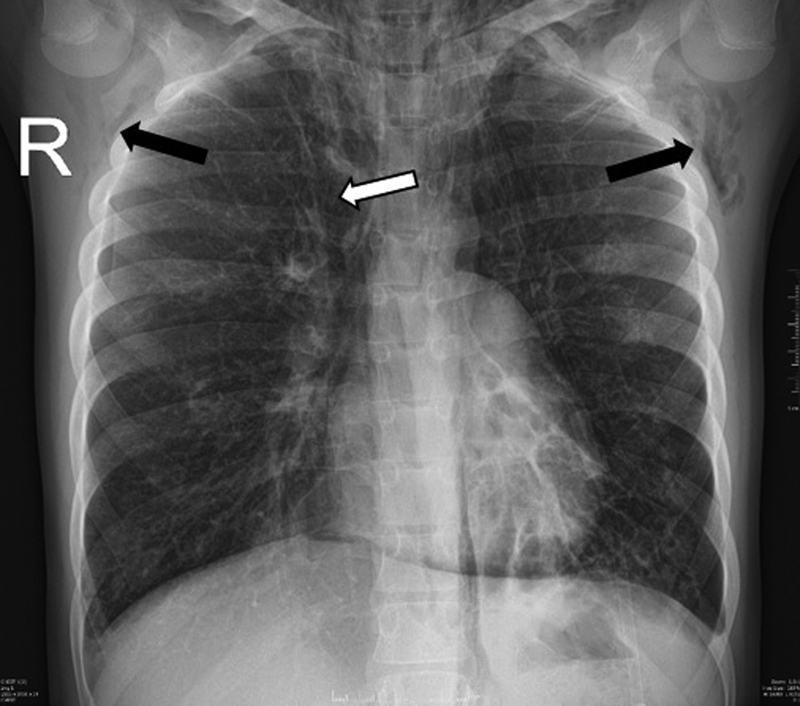
Chest X-ray, taken at the time of admission, also shows pneumomediastinum (white arrow); the subcutaneous emphysema is also visible (black arrows).

**Fig. 2:**
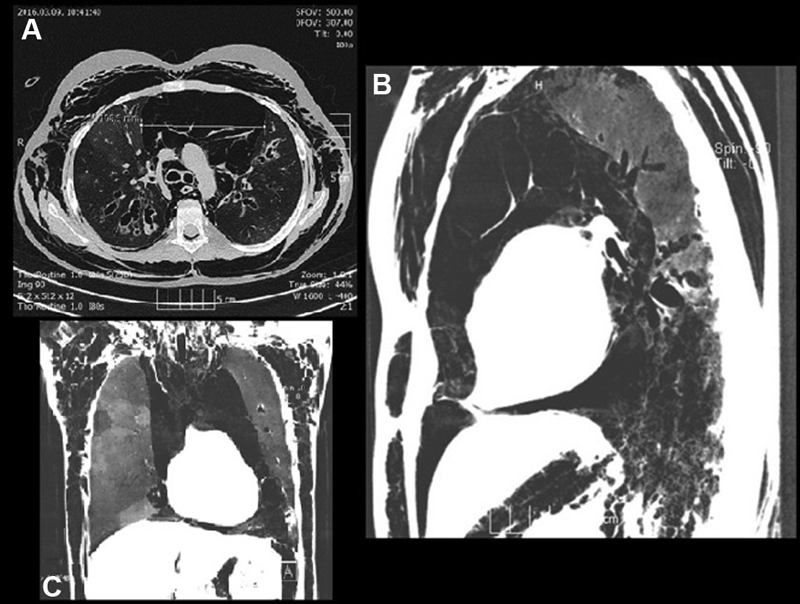
Computed tomography scan (in the level of bifurcation) depicted an extensive subcutaneous emphysema and a huge pneumomediastinum (diameter: 10.8 cm): ( **A** ) Transverse, ( **B** ) sagittal, and ( **C** ) coronal section.

**Fig. 3:**
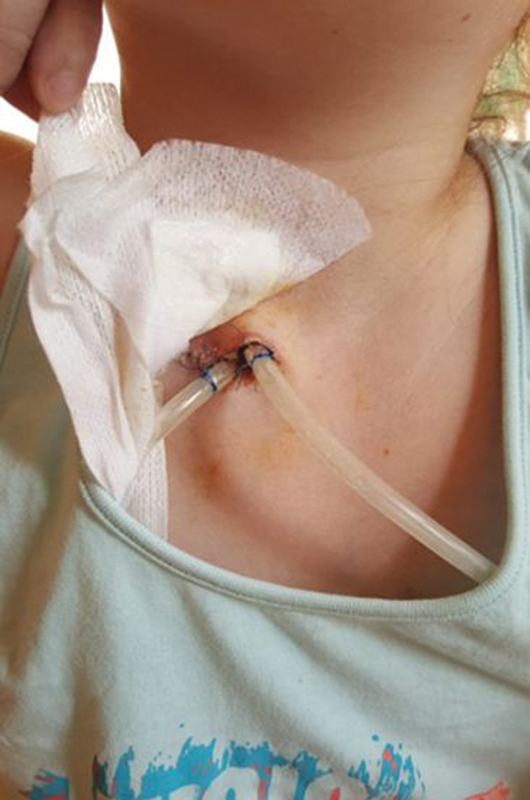
The drains leading to the mediastinum via the jugular notch.

**Fig. 4:**
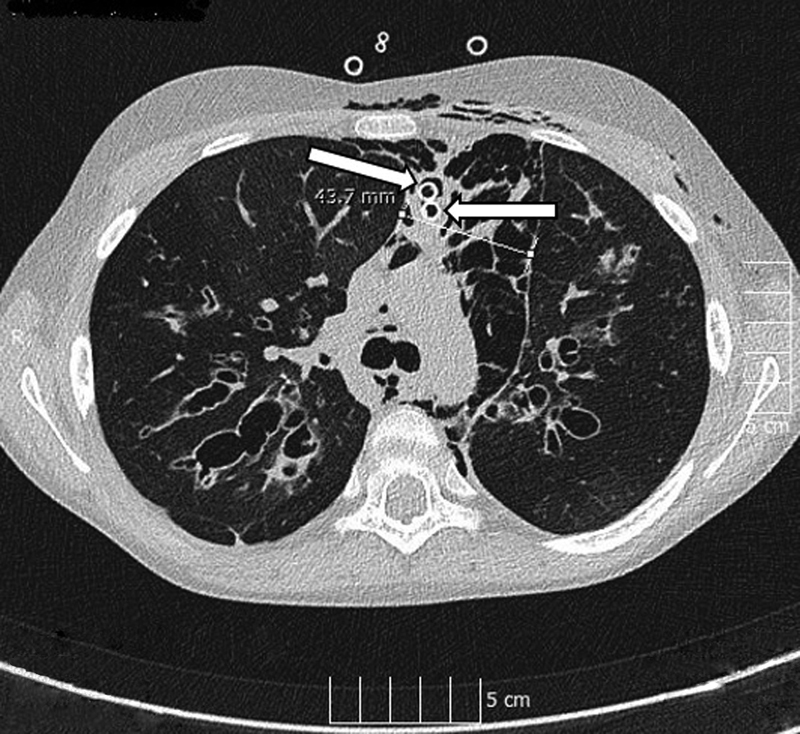
Control computed tomography scan (after 11 days of the first one) depicted a significant improvement (4.3 cm). The arrows show the mediastinal drains.

An 11-year-old girl, who was regularly followed up with CF, presented to our intensive care unit with an extended subcutaneous emphysema (left-sided facial, neck, upper third of the chest). CF was diagnosed during the neonatal period after surgical treatment of her meconium ileus. A day before her neck became suddenly painful, her face swelled and she mentioned severe thoracic pain associated with coughing. On auscultation, decreased breath sounds and quiet heart sounds were noticed. There was no shortness of breath; however, she was mildly tachypneic with transcutaneous oxygen saturations in the low 80%. She required oxygen support through nasal prongs to maintain her oxygen saturation above 90%. The chest X-ray showed pneumomediastinum ( Fig. 1 .). On the third day of hospitalization, she became tachypneic again; her oxygen requirement and the size of subcutaneous emphysema increased (neck, chest, and abdominal circumference increased, subcutaneous emphysema became palpable on the arms and the whole trunk); she felt like “a pillow pushing her back.” An urgent chest CT scan was performed, which showed extensive subcutaneous emphysema and a huge pneumomediastinum ( Fig. 2 ). She suddenly developed increased respiratory distress, tachycardia, and severe thoracic pain; therefore, an emergency needle mediastinal thoracocentesis was done in the second intercostal space at the midclavicular line on the left side of the thorax with application of local anesthesia. She immediately felt better but this intervention resulted in temporary improvement only. Based on the CF, the possibility of lung injury as a triggering mechanism was raised and discussed with the surgical team. As an emergency procedure, two mediastinal drains were placed through the jugulum under general anesthesia ( Fig. 3 ) that were put to continuous suction with 5 to 10 H _2_ O cm. Her symptoms significantly improved after a few hours and the subcutaneous emphysema decreased considerably. On the first postoperative day, the control chest X-ray described a 2 to 2.5 cm mantle PTX; therefore, a left-sided chest drain was inserted. Her general condition slowly improved, the subcutaneous emphysema decreased gradually, and the PTX resolved. Eleven days later, the chest CT scan was repeated ( Fig. 4 ), which showed a significantly smaller pneumomediastinum. At that time, a Heimlich valve was connected to the mediastinal drains, and after 5 days, on the 26th postoperative day, the drains were removed. The patient was transferred to our pulmonology ward, where, in addition to gradually restored inhalation treatments, mucus dissolution, anti-inflammatory therapy, and physiotherapy were commenced. Her oxygen requirement gradually decreased and she was discharged home after 48 days of in-hospital care.

## Discussion

The occurrence of SPM is a rare condition in childhood. 3 The majority of cases usually heal spontaneously, requiring only close observation, oxygen support, and bed rest. 1 3 5 6 7 8 9 Usually, the precipitating factors would include asthma, blunt thoracic trauma, or surgery in the region of the esophagus/trachea. 3 However, when the extent of pneumomediastinum is large, it can compromise respiration leading to the need for acute intervention. It is a literary rarity and can be observed mainly in traumatic event. This intervention involves emergency needle thoracocentesis. The authors were able to find only a few cases in the literature, where surgical intervention was needed. 3 With this case report, the authors wanted to illustrate that a life-threatening condition can develop without a traumatic event. In similar situations, sometimes a needle thoracocentesis is not always enough to resolve the symptoms. When the symptoms persist and the respiratory distress is increasing, a mediastinal (surgical) drainage is recommended through the jugulum. After introduction of the drains, active suction is necessary.

Some authors recommend bronchoscopy in patients with SPM, when tracheal injury is suspected. 10 We did not perform this diagnostic possibility, because it was an urgent situation requiring application of decompression. Furthermore, the repeated CT images using virtual bronchoscopy did not reveal any signs of bronchial or tracheal injury.

In patients with CF, the lung involvement determines the prognosis. Recurrent pneumonias, bronchiectasis, hemoptysis, and PTX are the main pulmonary complications. The duration of hospital stay was long with this patient, which was mainly due to the pulmonary complications of CF.

Here, the authors describe an association between CF and SPM. Our learning point for the future is in any patient with CF who presents in respiratory distress; together with findings of subcutaneous emphysema, it is necessary to exclude SPM.

In conclusion, the observation of patients as an inpatient with SPM is highly recommended, especially with underlying diseases. Occasionally, in these patients a life-threatening respiratory arrest can develop that would require urgent surgical intervention, such as mediastinal drain insertion. Introducing a thoracic drain through the jugular notch is both more comfortable for the patient and an easier option for the surgeon.

## Notes

New Insights and the Importance for the Pediatric Surgeon In severe spontaneous pneumomediastinum accompanied by respiratory distress, an urgent mediastinal drainage is recommended. The drain insertion is possible through the jugular notch.

## References

[1] Fitzwater J W Silva N N Knight C G Malvezzi L Ramos-Irizarry C Burnweit C A Management of spontaneous pneumomediastinum in children J Pediatr Surg 2015 50 06 983 986 25840604 10.1016/j.jpedsurg.2015.03.024

[2] Johnson J N Jones R Wills B K Spontaneous pneumomediastinum West J Emerg Med 2008 9 04 217 218 19561749 PMC2672281

[3] Chalumeau M Le Clainche L Sayeg N Spontaneous pneumomediastinum in children Pediatr Pulmonol 2001 31 01 67 75 11180677 10.1002/1099-0496(200101)31:1<67::aid-ppul1009>3.0.co;2-j

[4] Clinical Practice Guidelines for Pulmonary Therapies Committee Cystic Fibrosis Foundation Pulmonary Therapies Committee Flume P A Mogayzel P J Jr Robinson K A Rosenblatt R L Quittell L Marshall B C Cystic fibrosis pulmonary guidelines: pulmonary complications: hemoptysis and pneumothorax Am J Respir Crit Care Med 2010 182 03 298 306 20675678 10.1164/rccm.201002-0157OC

[5] Benlamkaddem S Berdai M A Labib S Harandou M A case of spontaneous pneumomediastinum with subcutaneous emphysema in children Children (Basel) 2018 5 02 22 10.3390/children5020022 PMC5835991 29414895

[6] Gerazounis M Athanassiadi K Kalantzi N Moustardas M Spontaneous pneumomediastinum: a rare benign entity J Thorac Cardiovasc Surg 2003 126 03 774 776 14502153 10.1016/s0022-5223(03)00124-7

[7] Bakhos C T Pupovac S S Ata A Fantauzzi J P Fabian T Spontaneous pneumomediastinum: an extensive workup is not required J Am Coll Surg 2014 219 04 713 717 25053221 10.1016/j.jamcollsurg.2014.06.001

[8] Tortajada-Girbés M Moreno-Prat M Ainsa-Laguna D Mas S Spontaneous pneumomediastinum and subcutaneous emphysema as a complication of asthma in children: case report and literature review Ther Adv Respir Dis 2016 10 05 402 409 27585598 10.1177/1753465816657478 PMC5933618

[9] Gasser C R Pellaton R Rochat C P Pediatric spontaneous pneumomediastinum: narrative literature review Pediatr Emerg Care 2017 33 05 370 374 26855340 10.1097/PEC.0000000000000625

[10] Gáti N Kassai T Prokopp T Pediatric tracheal injuries: report on 5 cases with special view on the role of bronchoscopy and managementInjury2020 10.1016/j.injury.2020.02.057 32067775

